# Real-time phase-contrast flow cardiovascular magnetic resonance with low-rank modeling and parallel imaging

**DOI:** 10.1186/s12968-017-0330-1

**Published:** 2017-02-10

**Authors:** Aiqi Sun, Bo Zhao, Yunduo Li, Qiong He, Rui Li, Chun Yuan

**Affiliations:** 1 Center for Biomedical Imaging Research, Department of Biomedical Engineering, School of Medicine, Tsinghua University, Haidian District, Beijing, China; 2 Athinoula A. Martinos Center for Biomedical Imaging, Massachusetts General Hospital, Chalestown, MA USA; 3 Department of Radiology, Harvard Medical School, Boston, MA USA; 4 Vascular Imaging Lab, Department of Radiology, University of Washington, Seattle, WA USA

**Keywords:** Cardiovascular imaging, Phase-contrast CMR, Cine, Real-time flow imaging, Model-based reconstruction, Low-rank modeling, Parallel imaging

## Abstract

**Background:**

Conventional phase-contrast cardiovascular magnetic resonance (PC-CMR) employs cine-based acquisitions to assess blood flow condition, in which electro-cardiogram (ECG) gating and respiration control are generally required. This often results in lower acquisition efficiency, and limited utility in the presence of cardiovascular pathology (e.g., cardiac arrhythmia). Real-time PC-CMR, without ECG gating and respiration control, is a promising alternative that could overcome limitations of the conventional approach. But real-time PC-CMR involves image reconstruction from highly undersampled (k*, t*)-space data, which is very challenging. In this study, we present a novel model-based imaging method to enable high-resolution real-time PC-CMR with sparse sampling.

**Methods:**

The proposed method captures spatiotemporal correlation among flow-compensated and flow-encoded image sequences with a novel low-rank model. The image reconstruction problem is then formulated as a low-rank matrix recovery problem. With proper temporal subspace modeling, it results in a convex optimization formulation. We further integrate this formulation with the SENSE-based parallel imaging model to handle multichannel acquisitions. The performance of the proposed method was systematically evaluated in 2D real-time PC-CMR with flow phantom experiments and in vivo experiments (with healthy subjects). Additionally, we performed a feasibility study of the proposed method on patients with cardiac arrhythmia.

**Results:**

The proposed method achieves a spatial resolution of 1.8 mm and a temporal resolution of 18 ms for 2D real-time PC-CMR with one directional flow encoding. For the flow phantom experiments, both regular and irregular flow patterns were accurately captured. For the in vivo experiments with healthy subjects, flow dynamics obtained from the proposed method correlated well with those from the cine-based acquisitions. For the experiments with the arrhythmic patients, the proposed method demonstrated excellent capability of resolving the beat-by-beat flow variations, which cannot be obtained from the conventional cine-based method.

**Conclusion:**

The proposed method enables high-resolution real-time PC-CMR at 2D without ECG gating and respiration control. It accurately resolves beat-by-beat flow variations, which holds great promise for studying patients with irregular heartbeats.

**Electronic supplementary material:**

The online version of this article (doi:10.1186/s12968-017-0330-1) contains supplementary material, which is available to authorized users.

## Background

Over the past few decades, phase-contrast cardiovascular magnetic resonance (PC-CMR) has been developed into a powerful tool for quantification and visualization of blood flow dynamics in the heart and large vessels [1–5]. It has advanced the understanding and diagnosis of various cardiovascular diseases, such as atherosclerosis [6], aneurysms [7], and arteriovenous malformation [8]. Conventional PC-CMR [9, 10] employs electro-cardiogram (ECG) synchronized cine acquisitions with respiration control to acquire data from multiple cardiac cycles, from which averaged velocity maps are obtained. Although this approach has been widely used in biomedical research and clinical practice, it suffers from a number of well-known limitations. For example, it often requires periodic or quasi-periodic cardiac motion to ensure efficient data acquisition; rejection of data caused by irregular cardiac motion often leads to prolonged acquisition time. Additionally, due to its underlying assumption, this approach only obtains averaged flow information over multiple cardiac cycles, failing to resolve beat-by-beat flow variations associated with irregular cardiac motion (e.g., cardiac arrhythmia). Capturing physiological and/or pathological flow variabilities has long been an important goal of PC-CMR research [11–14].

Real-time PC-CMR [15, 16] without ECG gating and respiration control is a promising direction to address these limitations; however, it requires a much higher imaging speed, posing significant challenges for both data acquisition and image reconstruction. A number of techniques have been developed to advance real-time PC-CMR. For example, advanced acquisition methods, such as echo-planar [17, 18], radial [19, 20], and spiral [21–24] acquisition schemes, have been employed for real-time PC-CMR. In addition, real-time PC-CMR also benefits from accelerated data acquisitions. For example, with the emergence of parallel imaging, sensitivity encoding (SENSE) [25] and generalized autocalibrating partially parallel acquisitions (GRAPPA) [26] have been applied to real-time PC-CMR [27–32]. More recently, model-based reconstruction methods [33, 34] using regularized nonlinear inversion [35] have been developed, achieving 2D real-time flow imaging with a spatial resolution of 1.5 mm and a temporal resolution of 25.6 ms by jointly reconstructing a proton density map, a phase map, and a set of coil sensitivities.

In this work, we present a new model-based method for real-time PC-CMR with sparse sampling. It is based on the integration of a novel low-rank model with parallel imaging. With temporal subspace modeling, the proposed method yields a convex optimization problem, thereby enabling efficient computation. The proposed method achieves real-time PC-CMR without ECG gating and respiration control, and well resolves the beat-by-beat flow variations that cannot be obtained from the conventional cine method. Compared with state-of-the-art real-time PC-CMR techniques, it provides higher temporal resolution. The effectiveness of the proposed method has been systematically evaluated in 2D real-time PC-CMR using both phantom experiments and in vivo experiments. A preliminary account of this work was presented in [36, 37].

## Theory

Ignoring flow during readout time, the imaging equation for real-time PC-CMR can be modeled as follows:

1 $$ {d}_{v, i}\left(\mathbf{k}, t\right)={\displaystyle \int {S}_i\left(\mathbf{r}\right){\rho}_v\left(\mathbf{r}, t\right)}{e}^{- j2\pi \mathbf{k}\cdot \mathbf{r}} d\mathbf{r}+{\eta}_{v, i}\left(\mathbf{k}, t\right), $$

where *ρ*_*v*_(**r**, *t*) denotes the dynamic image associated with either the flow-compensated (i. e., *v* = 1) or flow-encoded image sequence (i. e., *v* = 2, ⋯, *N*_*v*_), *S*_*i*_(**r**) the sensitivity map for the *i* th receiver coil (*i* = 1, 2, ⋯, *N*_*c*_), and *d*_*v*,*i*_(**k**, *t*) and *η*_*v*,*i*_(**k**, *t*) respectively the (k*, t*)-space measured data and measurement noise. Here, the goal is to reconstruct *ρ*_*v*_(**r**, *t*) from the undersampled data {*d*_*v*,*i*_(**k**, *t*)}, and then calculate the velocity maps as $\mathrm{V}\left(\mathbf{r}, t\right)=\frac{\varDelta \phi \left(\mathbf{r}, t\right)}{\uppi}\cdot$VENC, where *Δϕ*(**r**, *t*) = ∠ *ρ*_*v*_(**r**, *t*) − ∠ *ρ*_1_(**r**, *t*) denotes the phase difference between the flow-encoded and flow-compensated image sequences, and VENC the pre-specified encoding velocity. Since in real-time PC-CMR, there is no data sharing with ECG gating, (k*, t*)-space data is often highly undersampled. Direct inversion of {*d*_*v*,*i*_(**k**, *t*)} can incur significant aliasing artifacts and lead to inaccurate velocity measurements.

Here we introduce a low-rank model-based reconstruction method with parallel imaging to address the problem. For convenience, we consider a discrete image model, in which each flow image sequence can be represented as a spatiotemporal Casorati matrix [38], i.e.,

2 $$ {\mathbf{C}}_v=\left[\begin{array}{ccc}\hfill {\rho}_v\left({\mathbf{r}}_1,{t}_1\right)\hfill & \hfill \cdots \hfill & \hfill {\rho}_v\left({\mathbf{r}}_1,{t}_M\right)\hfill \\ {}\hfill \vdots \hfill & \hfill \ddots \hfill & \hfill \vdots \hfill \\ {}\hfill {\rho}_v\left({\mathbf{r}}_N,{t}_1\right)\hfill & \hfill \cdots \hfill & \hfill {\rho}_v\left({\mathbf{r}}_N,{t}_M\right)\hfill \end{array}\right]\in {\mathbb{C}}^{N\times M}. $$

Similar to cardiac imaging applications [39–41], each **C**_*v*_ admits a low-rank approximation due to strong spatiotemporal correlation of time-series images. Moreover, due to the nature of flow encoding, there is also strong spatial and temporal correlation among different flow image sequences. To exploit such correlation, the following joint Casorati matrix is introduced:

3 $$ \mathbf{C}=\left[{\mathbf{C}}_1,\cdots, {\mathbf{C}}_{N_v}\right], $$

on which we enforce the low-rank structure, i.e., rank(**C**) ≤ *L*. There are a number of ways of imposing low-rank constraints [38, 40, 42, 43]. Here, we use an explicit rank constraint via matrix factorization, i.e., **C** = **UV**, where **U** ∈ *ℂ* ^*N* × *L*^ and **V** ∈ *ℂ* ^*L* × *M*^. In this low-rank representation, the columns of **U** and rows of **V** respectively span the spatial subspace and temporal subspace of **C**.

Next, we formulate the low-rank constrained reconstruction problem. First, note that with matrix-vector notation, Eq. (1) can be written as:

4 $$ {\mathbf{d}}_i=\Omega \left({\mathbf{F}}_{\mathrm{s}}{\mathbf{S}}_i\mathbf{C}\right)+{\mathbf{n}}_i, $$

where **d**_*i*_ denotes the measured data, Ω the sparse sampling operator, **F**_s_ the spatial Fourier transform matrix, and **S**_*i*_ and **n**_*i*_ respectively the sensitivity map and measurement noise. Imposing the low-rank constraint, the image reconstruction problem can be formulated as

5 $$ \left\{\widehat{\mathbf{U}},\widehat{\mathbf{V}}\right\}= \arg \underset{\left\{\mathbf{U},\mathbf{V}\right\}}{ \min }{\displaystyle \sum_{i=1}^{N_c}{\left\Vert {\mathbf{d}}_i-\Omega \left[{\mathbf{F}}_{\mathrm{s}}{\mathbf{S}}_i\left(\mathbf{UV}\right)\right]\right\Vert}_2^2}. $$

This problem is a non-convex optimization problem, for which a number of algorithms can be applied (e.g., [44, 45]).

**Fig. 1:**
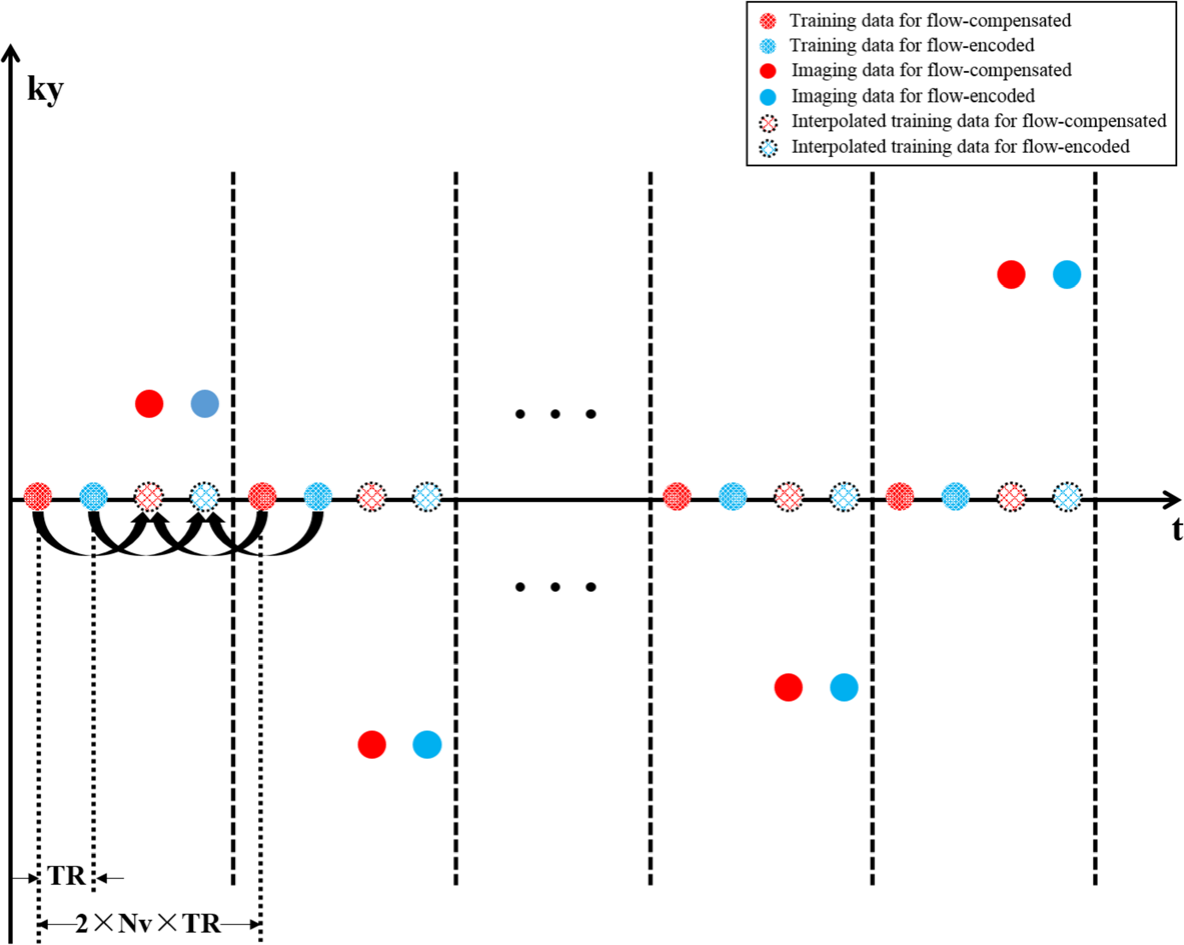
The proposed (k*, t*)-space sampling scheme. Here the temporal training data are acquired from the central k-space, while the imaging data are acquired from the outer k-space. The same sampling pattern is applied for the flow-compensated and flow-encoded data sets. Temporal interpolation is performed to ensure the training data are at the time instants as the imaging data. Note that with this sampling scheme, the (nominal) temporal resolution is 2 × *N*_*v*_ × *TR*

The image reconstruction problem can be further simplified. Extending the early work in cardiac imaging [38, 40, 41, 46], we can pre-estimate the temporal subspace **V** by acquiring training data with a specialized data acquisition scheme. Specifically, as shown in Fig. 1, we design an interleaved sampling pattern, in which both training data and imaging data are collected. Here, the training data are sampled from the central k-space, while the imaging data are acquired from the remaining (k*, t*)-space region with a random sampling scheme. With this sampling scheme, the two sets of data provide the complementary information for the low-rank model: the training data have high temporal resolution, while the imaging data have high spatial resolution. From the training data, we estimate the temporal subspace using the principal component analysis [38, 47]. With the imaging data, we estimate the spatial subspace **U**. To match the timing between the two sets of data, a proper temporal interpolation is performed, which interpolates the training data into those at the same time instants as the imaging data. Note that with such a scheme, the temporal resolution for the proposed method is 2 × *N*_*v*_ × *TR*. Moreover, note that the coil sensitivities **S**_*i*_ can be estimated from temporal averaged (k*, t*)-space data from the flow-compensated image sequence.

With $\widehat{\mathbf{V}}$, we can determine **U** by solving the following convex optimization problem:

6 $$ \widehat{\mathbf{U}}= \arg \underset{\mathbf{U}\in {\mathbb{C}}^{N\times L}}{ \min }{\displaystyle \sum_{i=1}^{N_c}{\left\Vert {\mathbf{d}}_i-\Omega \left[{\mathbf{F}}_{\mathrm{s}}{\mathbf{S}}_i\left(\mathbf{U}\widehat{\mathbf{V}}\right)\right]\right\Vert}_2^2}. $$

**Fig. 2:**
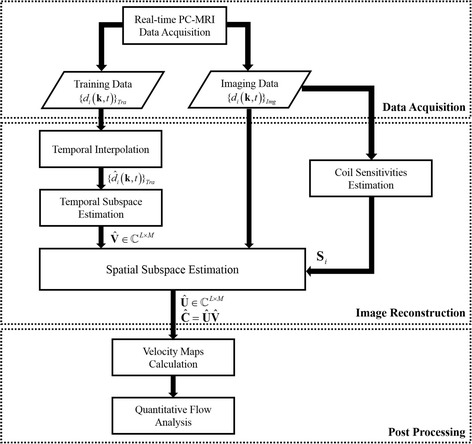
The data processing pipeline for the proposed real-time PC-CMR method. This pipeline consists of three major components: data acquisition, image reconstruction, and post processing

Due to the temporal subspace estimation, the low-rank matrix recovery problem has been reduced to a simple least-squares problem. By solving **Û**, the joint Casorati matrix can be reconstructed as $\widehat{\mathbf{C}}=\widehat{\mathbf{U}}\widehat{\mathbf{V}}$, from which we can obtain each flow image sequence and estimate the flow velocities. A diagram summarizing the proposed method is shown in Fig. 2.

## Methods

We performed both phantom and in vivo studies to evaluate the performance of the proposed method for 2D real-time PC-CMR. The experiments were conducted on a 3.0 T whole body MR scanner (Achieva, Philips Medical System, Best, The Netherlands), equipped with a 32-channel cardiovascular coil. A gradient-echo (GRE) based pulse sequence was adapted to implement the proposed real-time acquisition scheme as shown in Fig. 1. Here neither ECG gating nor respiration control was used to aid data acquisition. Additionally, we performed conventional cine PC-CMR using a vendor-provided GRE-based pulse sequence, in which retrospective ECG gating was used.

**Fig. 3:**
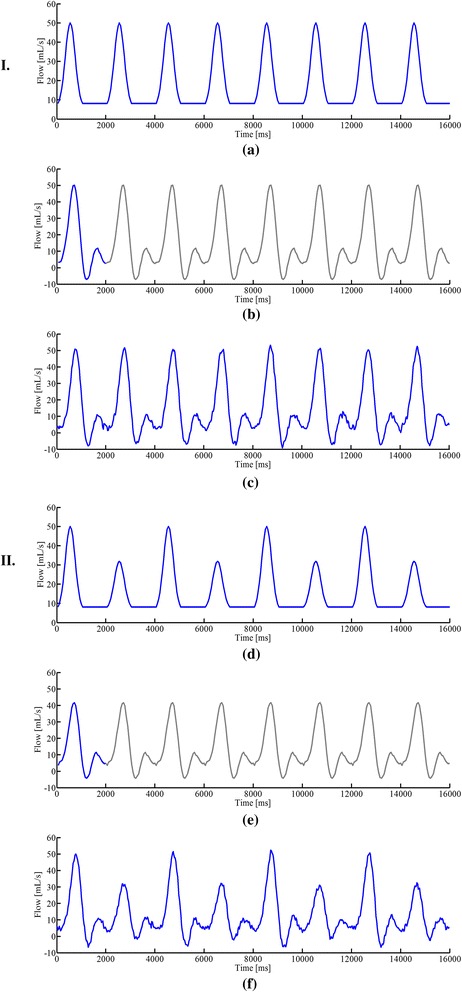
Reconstructed flow waveforms for the phantom experiment. **a** Pre-designed flow waveform (**I**) and reconstructed flow waveforms from the cine imaging method (**b**) and the proposed real-time imaging method (**c**). **d** Pre-designed flow waveform (**II**) and reconstructed flow waveforms from the cine imaging method (**e**) and the proposed real-time imaging method (**f**). Note that we manually repeated the cine flow waveforms in (**b**) and (**e**) with the gray color, which should facilitate the comparison with the proposed real-time imaging method. Here the flow waveforms from both the cine method and the real-time imaging method exhibit some discrepancy with the input waveform for the programmable pump during the periods of constant flow. This may be caused by the reflected bell-shape flow after it hits the wall of the tube

First, flow phantom experiments were performed to evaluate the capability of the proposed method in resolving various flow dynamics. Specifically, a 15-mm-diameter plastic tube simulating large vessel in the aorta was filled with blood-mimicking fluid [48], and plugged into a container (filled with water and positioned in the magnetic isocenter along the z-direction). The tube was further connected with a computer-programmable pump (CompuFlow 5000 MR, Toronto, Canada) [49], with which we can set up different flow waveforms for the phantom experiments. Here, the two flow waveforms were used: flow waveform (I), as shown in Fig. 3a, repeating at a 2 s period within which a 1 s bell-shape flow is followed by a 1 s constant flow; and flow waveform (II), as shown in Fig. 3d, repeating at a 4 s period within which two different 1 s bell-shape flows are separated by a constant flow. To obtain flow measurements, we performed a one-directional velocity encoding along the foot-head (FH) direction for both cine and real-time experiments. For cine flow imaging, we assumed that the heart beat period is 2 s for ECG gating. Under this assumption, the waveform (I) represents a periodic flow, whereas the waveform (II) represents aperiodic flow. For both cine and real-time flow experiments, we used the following imaging parameters: field of view (FOV) = 220 mm *×* 120 mm, matrix size = 182 *×* 100, spatial resolution = 1.20 mm *×* 1.20 mm, slice thickness = 5 mm, repetition time (TR) = 5.0 ms, echo time (TE) = 3.0 ms, flip angle = 10°, and VENC = 100 cm/s. Notice that the temporal resolution for the real-time acquisition is 4 *×* TR = 20 ms, while, for the cine acquisition, the temporal resolution is 56 ms (with 36 cardiac phases). The total acquisition time was around 42 s for both experiments.

Second, in vivo experiments were performed to evaluate the proposed method. Ten healthy volunteers (7 males, age: 22–29 years, median: 25 years), who had no symptoms of cardiovascular diseases, were recruited. In addition, we performed a feasibility study of applying the proposed method for arrhythmia detection, and recruited two patients (2 males, age: 23-year old and 72-year old). This study was approved by the Institutional Review Board at Tsinghua University, and all the subjects gave written informed consent. Both the cine and real-time flow experiments were performed on the planes perpendicular to the ascending aorta (AAo) and descending aorta (DAo) during free breathing, and with one directional velocity encoding along the FH direction. For the cine acquisition, the retrospective ECG gating was set according to an estimate of each subject’s heartbeat period, and three averages were performed to mitigate respiratory motion artifacts. For both the cine and real-time imaging experiments, the following imaging parameters were used: FOV = 240 mm × 225 mm, matrix size = 132 × 124, spatial resolution = 1.80 mm *×* 1.80 mm, slice thickness = 5 mm, TR/TE = 4.5/2.8 ms, flip angle = 10°, and VENC = 200 cm/s. For the real-time flow imaging, the temporal resolution is 4 × TR = 18 ms, whereas for the cine imaging, the temporal resolution is around 36 ms (with 28 cardiac phases). The total acquisition time was around 94 s for both experiments.

For cine flow imaging, the flow-compensated and flow-encoded images were simply reconstructed from the fully-sampled data. For the proposed real-time flow imaging, we followed the procedure illustrated in Fig. 2. Specifically, we first performed the temporal interpolation and estimated the temporal subspace **V** from the training data. We then estimated the coil sensitivity maps **S**_*i*_ from the temporally averaged (k*, t*)-space measurements. We further determined the spatial subspace **U** by solving Eq. (6), followed by forming the time-series images for flow-compensated and flow-encoded images. To improve the computational efficiency, proper coil compression (e.g., [50]) can be adopted. After image reconstruction, phase correction [51] was performed to correct the phase offsets caused by eddy currents. The velocity maps were then extracted for quantitative flow analysis.

We analyzed the results of the phantom and in vivo experiments. For the phantom experiments, the flow waveforms obtained from the cine and real-time flow imaging methods were analyzed for both periodic and aperiodic flow patterns. For the in vivo experiments with healthy subjects, we evaluated the degree of agreement between the flow measurements from the cine method and those from the proposed method. Specifically, we performed a Bland-Altman analysis, as well as a paired Student’s *t*-test, on the peak velocities and stroke volumes obtained from the two methods. Here the peak velocity is defined as the maximum velocity within one cardiac cycle, and the stroke volume is the integral of the flow velocity over one cardiac cycle within the ascending aorta. For the experiments with arrhythmic patients, we evaluated the flow variabilities captured by the proposed method with reference to an external ECG recording of cardiac motion.

To evaluate the effectiveness of imposing a low-rank constraint on the joint Casorati matrix $$ \mathbf{C}=\left[\begin{array}{cc}\hfill {\mathbf{C}}_1\hfill & \hfill {\mathbf{C}}_2\hfill \end{array}\right] $$, we performed a comparison with an alternative formulation, in which the low-rank constraint is enforced for each individual flow image sequence. The signal-to-noise (SNR) and velocity-to-noise (VNR) were calculated for the magnitude images and velocity maps, respectively. Here SNR was calculated as a ratio between the mean signal intensity over a region of interest (ROI) and the standard deviation of the background, whereas VNR was calculated as a ratio between the mean velocity for the same ROI and the standard deviation for a region in the stationary tissue [52].

## Results

Representative results are shown to illustrate the performance of the proposed method. Figure 3 shows the flow waveforms for the phantom experiments obtained from the conventional cine method and the proposed real-time imaging method. Here the input flow waveforms for the pump were also shown. As can be seen, for the flow waveform (I) (i.e., periodic flow), both the cine and real-time imaging methods can capture the flow dynamics. In particular, the peak flows obtained from the two methods were accurate. However, for the flow waveform (II) (i.e., aperiodic flow), only the proposed method resolves the significant flow variations. The conventional cine method, which integrates data into a single cardiac cycle, fails to reconstruct the aperiodic flow dynamics (e.g., erroneous peak flows).

**Fig. 4:**
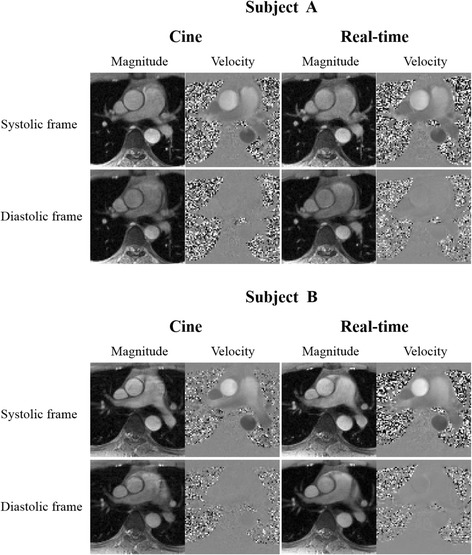
Comparisons of real-time flow imaging with cine flow imaging for two healthy subjects. The magnitude images and velocity maps respectively from conventional cine method and the proposed real-time flow imaging method are shown

Figure 4 shows the in vivo results for two healthy subjects. Here, we show the reconstructed magnitude images and velocity maps corresponding to a systolic cardiac phase and a diastolic cardiac phase. As can be seen, the proposed method provides at least comparable reconstruction quality to the cine method. Although both methods can resolve the vessel structure, the real-time imaging method is more motion-robust than the cine method. To better illustrate the proposed method, a reconstruction video for one healthy subject was included (see Additional file 1).

**Fig. 5:**
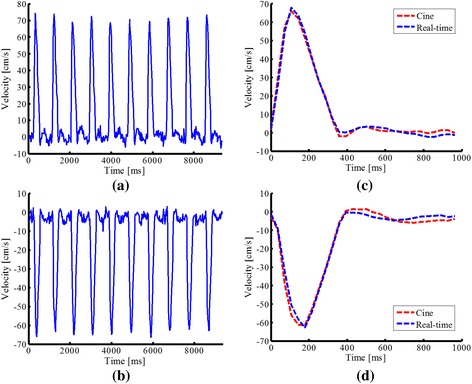
Reconstructed velocity waveforms from the proposed method for a healthy subject. The velocity waveforms associated with the ascending aorta (AAo) and descending aorta (DAo) over 10 cardiac cycles are shown in (**a**) and (**b**). The averaged flow velocities over 30 consecutive cardiac cycles from the proposed real-time flow imaging method are compared with the ones from the cine method for both AAo (**c**) and DAo (**d**). Here, the averaging is performed as follows. We first segment the reconstructed velocity waveforms from the proposed method into sub-waveforms, each of which corresponds to a single cardiac cycle. Second, we average these sub-waveforms to obtain a synthetic flow waveform for one cardiac cycle. If a heartbeat period is different from the one in the cine method, temporal interpolation is performed

In addition, we analyzed the mean flow velocities associated with two ROIs in AAo and DAo. Figure 5a and b respectively show the velocity waveforms over 10 consecutive cardiac cycles for a healthy subject. Clearly, the proposed method well resolves beat-by-beat variations. We further evaluated how the velocity waveforms from the real-time imaging are related to those from the conventional cine method. We averaged the velocity waveforms over 30 consecutive cardiac cycles from the proposed method into one velocity waveform associated with a synthetic cardiac cycle, and then compared it with that from the cine method. From Fig. 5c and d, it is evident that the averaged velocity waveforms for AAo and DAo correlate well with those from the conventional cine method. In particular, both methods yield very similar peak velocities for the AAo and DAo.

**Fig. 6:**
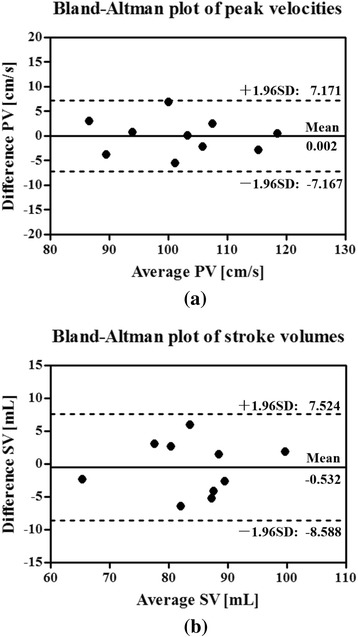
Bland-Altman analysis. Bland-Altman analysis of peak velocities (**a**) and stroke volumes (**b**) comparing the proposed real-time imaging method with the conventional cine method. The peak velocities and stroke volumes from real-time imaging are the mean values over 30 consecutive cardiac cycles. In the above plots, the central solid horizontal line indicates the mean of the differences in the measurements from two methods, while the outer dotted horizontal lines indicate the lower/upper limits of agreement

We also performed a statistical analysis of the results from the two methods for all ten healthy subjects. Figure 6a and b respectively show the Bland-Altman plots of peak velocities and stroke volumes that compare the two methods. As can be seen, the results from the proposed method are in excellent agreement with those from the conventional cine method. In addition, we performed the paired Student’s *t*-test analysis on the two methods, and the correlation coefficients for peak velocities and stroke volumes are 0.94 (*P <* 0*.*0001) and 0.90 (*P* = 0*.*0002), respectively. This further confirms strong correlation between the two methods.

**Fig. 7:**
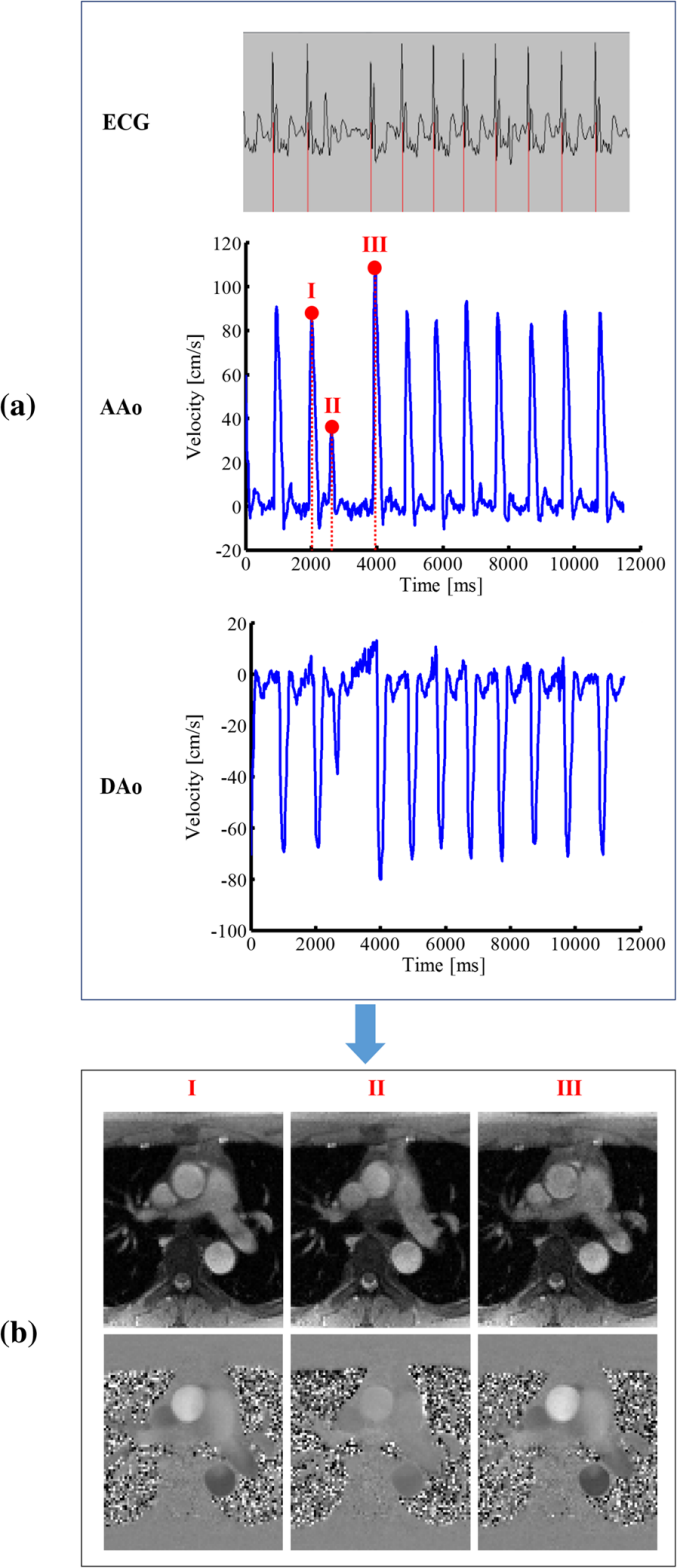
Real-time PC-CMR for the 23-year-old arrhythmic patient. **a**: The ECG recordings and the velocity waveforms of AAo and DAo. **b**: The magnitude images and velocity maps for the three representative time frames within an arrhythmic period. As can be seen, the proposed method nicely captures a dramatic change of flow velocities occurring during an arrhythmia period. Note that this type of flow dynamics cannot be obtained from the conventional cine method

Figure 7 shows the reconstruction results for the 23-year-old patient (with mild cardiac arrhythmia). As expected, the proposed method is able to reconstruct flow variations over different cardiac cycles. In particular, as shown in Fig. 7b, the proposed method nicely captures a sudden flow velocity drop occurring in an arrhythmic period. Note that this type of flow dynamics cannot be obtained from the conventional cine method. Further, it is worth noting that the flow velocity variations correlate well with the ECG signal recorded during the acquisition. Besides, we show three snapshot images from the proposed method. Clearly, the velocity maps confirm the dramatic flow variations within the arrhythmic period.

**Fig. 8:**
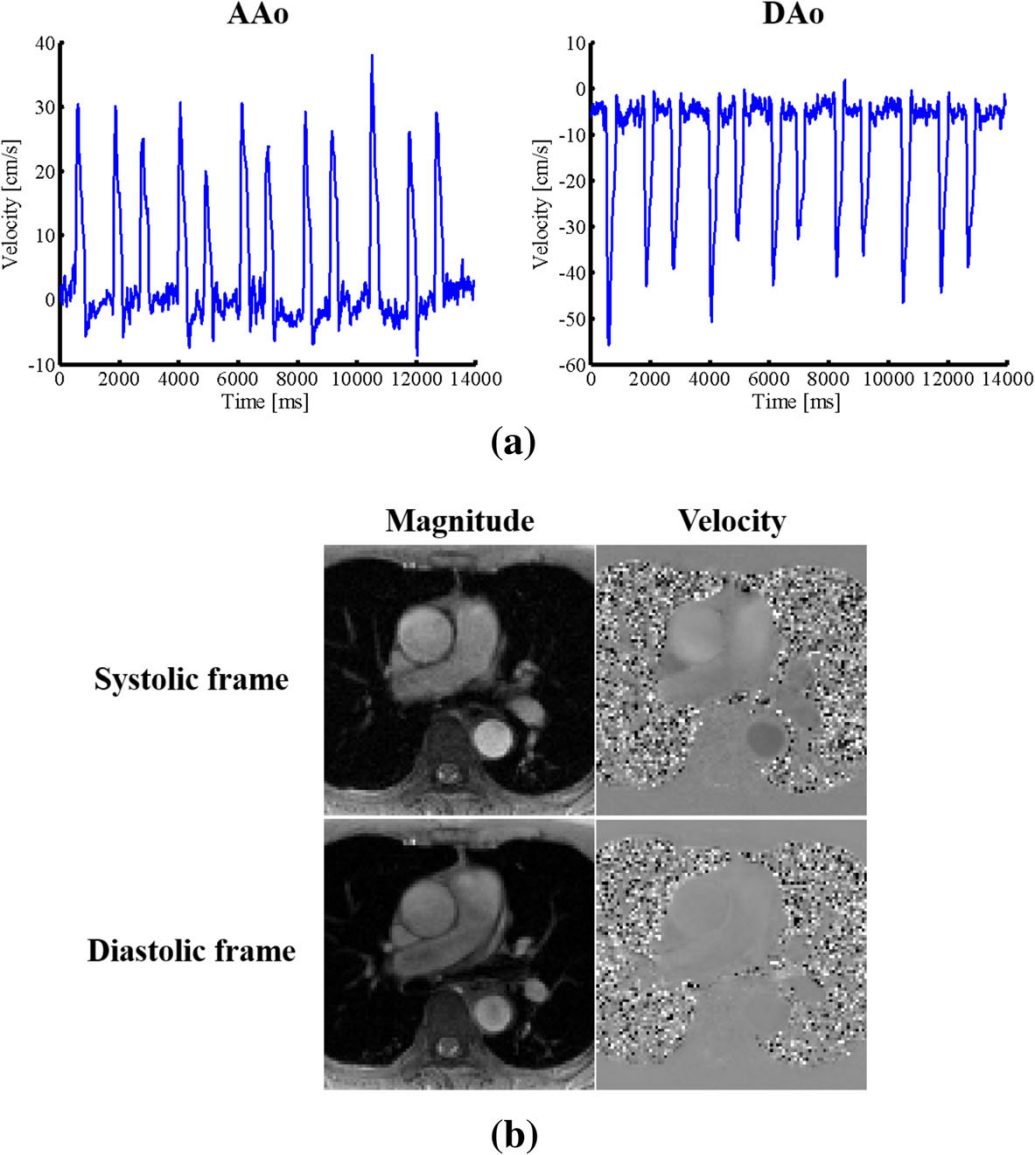
Real-time PC-CMR for the 72-year-old arrhythmic patient. **a**: The velocity waveforms associated with the AAo and DAo over 12 consecutive cardiac cycles from the proposed method. **b**: Reconstructed magnitude images and velocity maps corresponding to a systolic cardiac frame and a diastolic cardiac frame

Figure 8 shows the reconstruction results for the 72-year-old patient (with severe cardiac arrhythmia). The velocity waveforms associated with the AAo and DAo from the proposed method are shown in Fig. 8a. Again, the proposed method well captures irregular flow variations, which are more significant than the ones from the previous patient. Moreover, we show the reconstructed magnitude images and velocity maps in Fig. 8b, and include the corresponding reconstruction video in Additional file 2.

**Fig. 9:**
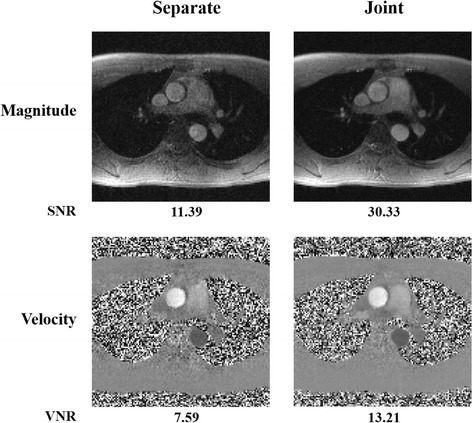
Comparisons of joint low-rank reconstruction with separate reconstruction. The magnitude images and velocity maps from the proposed joint reconstruction method are compared with the results from the separate method. The results were reconstructed by the two methods using the same data set (i.e., a 40 s PC-CMR acquisition) acquired from a healthy subject. Both the methods applied the same rank value (i.e., *L* = 20). The corresponding reconstruction signal-to-noise ratio (SNR) for the magnitude image and velocity-to-noise ratio (VNR) for the velocity map are shown under each image

Figure 9 compares the magnitude images and velocity maps from the proposed method using the joint low-rank constraint with that using the separate low-rank reconstruction. Here the two methods reconstructed the same data set (i.e., a 40 s real-time PC-CMR acquisition), and used the same rank value *L* = 20. As can be seen, the proposed method reconstructs the spatial images and velocity maps with improved quality over the alternative formulation. This illustrates the benefits of imposing the low-rank constraint on the joint Casorati matrix.

## Discussion

In this work, we introduced a new real-time flow imaging method and systematically demonstrated its effectiveness with both flow phantom experiments and in vivo experiments. Here, it is worth reiterating the key characteristics of the proposed method. First, the proposed method can be used as a viable alternative to the conventional cine flow imaging method in that it provides comparable (if not superior) image quality and flow information for healthy subjects. Second, the proposed method is able to resolve beat-by-beat physiological and/or pathological flow variations, which cannot be obtained from the conventional cine method based on ECG gating and respiration control. Such information is often clinically important (e.g., for assessing cardiac arrhythmia).

As with other model-based methods, the proposed method involves model selection (i.e., selection of the rank *L*). Generally, the selection of *L* needs to balance the model representational power, the number of measurements (i.e., acquisition time), and signal-to-noise ratio [40]. In this work, we manually selected *L* to trade off the above factors, and it consistently yielded good reconstruction performance, although it is worthwhile to investigate other principled model selection methods (e.g., [53, 54]) in future research.

The proposed formulation results in a convex optimization problem, which enables efficient computation. For example, the runtime for reconstructing an in vivo dataset (from 94 s real-time acquisition) takes around 10 min on a workstation with 64 GB RAM and 3.47 GHz CPU. The computational efficiency may be further improved by an implementation on graphical processing units. Such an investigation is beyond the scope of this paper, but is worthwhile to explore for future research.

In addition to rank constraint, sparsity constraint can also be incorporated to accelerate PC-CMR. It has been demonstrated in [40, 43, 55] that joint low-rank and sparsity constrained reconstruction leads to improved performance for dynamic MRI. Along this line, we can extend the proposed real-time flow imaging method by exploiting our early work [56] in cine flow imaging, although such an extension will come with additional computational cost.

The flow-compensated and flow-encoded images share similar magnitude but different phase differences. We can extend the proposed method to exploit such information and impose a stronger constraint in the model-based reconstruction. However, the resulting formulation can involve a joint reconstruction of magnitude and phase images, which generally leads to a non-convex optimization problem. To solve such a problem, specialized algorithms and proper initialization are often needed. In contrast, the proposed method here employs a low-rank model to exploit the spatiotemporal correlation between flow images, which leads to a simple convex problem formulation and efficient computation. Given that the two models may have different trade-offs, comprehensively evaluating their advantages and drawbacks is a very interesting open problem to be explored in future work.

In this work, we demonstrate the performance of the proposed method for 2D real-time flow imaging, in which through-plane flow was imaged. Considering the complex flow patterns and blood vessel geometry, it is highly desirable to perform 3D real-time flow imaging. However, 3D real-time flow imaging generally involves a more challenging trade-off between spatial resolution, temporal resolution, and imaging time, and a significantly more challenging computational problem. We are investigating an extension of the proposed method to 3D real-time flow imaging, and the results will be reported in future work.

This paper is focused on the development of a novel real-time flow imaging technique, which should serve as a foundation for our subsequent clinical studies. Given that the proposed method well resolves beat-by-beat flow variations, it can provide more information on hemodynamics for patients with significant irregular heartbeats. In the future work, we plan to conduct systematic study of the proposed method for various potential clinical applications (e.g., atrial fibrillation, premature atrial contraction or congenital heart disease).

It is also worthwhile to remark on the potential limitations of the proposed method. First, note that the aforementioned spatial and temporal resolution both refer to nominal resolution. For a linear shift-invariant reconstruction method (e.g., conventional Fourier reconstruction), the resolution can be characterized through the point spread function. However, for a nonlinear reconstruction method (e.g., sparsity [57] or low-rank constrained reconstruction [38, 40]), rigorously characterizing the resolution has been a long-standing open problem. In this work, we turn to reporting the nominal spatial and temporal resolution, although it is worthwhile to perform an in-depth study of resolution characterization for these advanced image reconstruction methods in future research.

Second, it is useful to create gold standard data sets for studying real-time flow imaging. Due to the undersampling nature of real-time imaging experiments, it is often difficult to generate an ideal reference for systematic quantitative evaluation. For example, in the phantom experiments, the input flow waveforms for the pump deviate from the flow measurements during the constant flow due to the phantom response to the flow/pressure in the tubing system. In the future, we hope to build a more advanced flow imaging phantom, in which better reference data sets can be generated.

## Conclusions

A new model-based method was introduced for high-resolution real-time PC-CMR without ECG gating and respiration control. It integrates the novel low-rank model with parallel imaging, which enables high-quality image reconstruction from highly undersampled (k*, t*)-space data for real-time PC-CMR. The effectiveness and utilities of the proposed method have been demonstrated for 2D real-time PC-CMR with both phantom experiments and in vivo experiments. We expect that the proposed method will enhance the practical utility of real-time PC-CMR for various clinical applications.

## Additional files

Additional file 1: Real-time PC-CMR of a healthy subject. This video includes the reconstructed magnitude images and velocity maps by the proposed method for a healthy subject. (GIF 3557 kb)
Additional file 2: Real-time PC-CMR of an arrhythmic patient. This video includes the reconstructed magnitude images and velocity maps by the proposed method for an arrhythmic patient. (GIF 4693 kb)

## Abbreviations

AAo: Ascending aorta
DAo: Descending aorta
ECG: Electro-cardiogram
FOV: Field of view
GRAPPA: Generalized autocalibrating partially parallel acquisitions
PC-CMR: Phase-contrast cardiovascular magnetic resonance
SENSE: Sensitivity encoding
TE: Echo time
TR: Repetition time
VENC: Encoding velocity
