# Supplementary figures and images for: Real-time phase-contrast flow cardiovascular magnetic resonance with low-rank modeling and parallel imaging
Source: J Cardiovasc Magn Reson. 2017 Feb 10;19:19. doi: 10.1186/s12968-017-0330-1 (PMC5301411; doi:10.1186/s12968-017-0330-1)

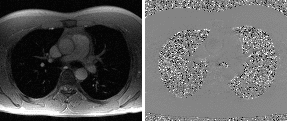

Supplement: Additional file 1: — Real-time PC-CMR of a healthy subject. This video includes the reconstructed magnitude images and velocity maps by the proposed method for a healthy subject. (GIF 3557 kb) [file 12968_2017_330_MOESM1_ESM.gif]

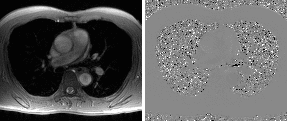

Supplement: Additional file 2: — Real-time PC-CMR of an arrhythmic patient. This video includes the reconstructed magnitude images and velocity maps by the proposed method for an arrhythmic patient. (GIF 4693 kb) [file 12968_2017_330_MOESM2_ESM.gif]
